# Aggressive Digital Papillary Adenocarcinoma

**Published:** 2015-11-09

**Authors:** Jake Laun, Jared Gopman, Connor W. Barnes, Kelly A. Segars, Joshua B. Elston, Jeffrey Stone

**Affiliations:** ^a^Division of Plastic Surgery, Department of Surgery, University of South Florida Morsani College of Medicine, Tampa; ^b^Largo Medical Center, Largo, Fla; ^c^Florida Orthopedic Institute, Tampa

**Keywords:** digital mass, hand, adenocarcinoma, papillary, adnexal carcinoma

## DESCRIPTION

**Figure 1:**
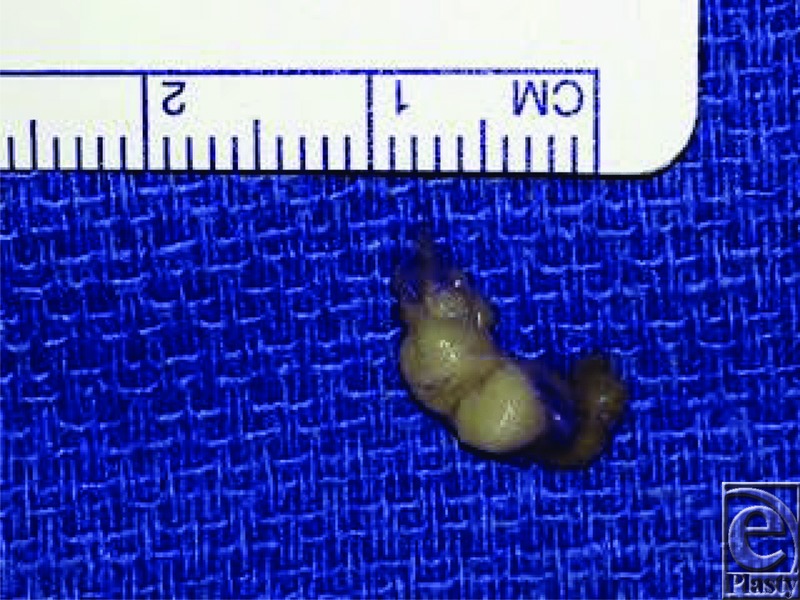
Gross specimen of left long finger mass after resection.

A 54-year-old man presented with a 7-year history of left long finger distal interphalangeal joint region mass. He underwent mass excision, and pathology demonstrated adenocarcinoma (Fig 1). The patient subsequently underwent an extensive oncological workup without identification of known primary source. Pathology stains confirmed primary aggressive digital papillary adenocarcinoma (ADPA).

## QUESTIONS

1. **What is an ADPA and how does it present?**
2. **How is ADPA diagnosed?**
3. **How is ADPA treated?**
4. **What is the prognosis after diagnosis of ADPA?**

## DISCUSSION

Primary cancers of the hand are most often due to squamous cell carcinoma or basal cell carcinoma and account for 10% to 15% of all skin malignancies.^1^^,^^2^ One rare primary cancer that is found in the digits is an ADPA. First described in 1979 as an “eccrine acrospiroma,” it was later described as an “aggressive digital papillary adenocarcinoma.”^3^ ADPA mainly occurs in men 50 to 70 years old and arises from sweat glands. It was originally thought to have arisen from a benign counterpart called an aggressive digital papillary adenoma due to histological differences. Clinically, ADPA may appear as a small, painless mass that enlarges over several years; however, they may also ulcerate and bleed. The most common locations are the volar surface or the skin between the nail bed and the distal interphalangeal joint, with almost 80% of cases in the digits of the upper extremity.^4^^,^^5^ They are often misdiagnosed, resulting in delayed treatment as they present similar to benign conditions such as a ganglion cyst, pyogenic granuloma, or soft-tissue infection.^4^ All cases are considered malignant tumors, as they are aggressive, recur often, metastasize to distant sites, and invade local tissues including bone.

**Figure 2:**
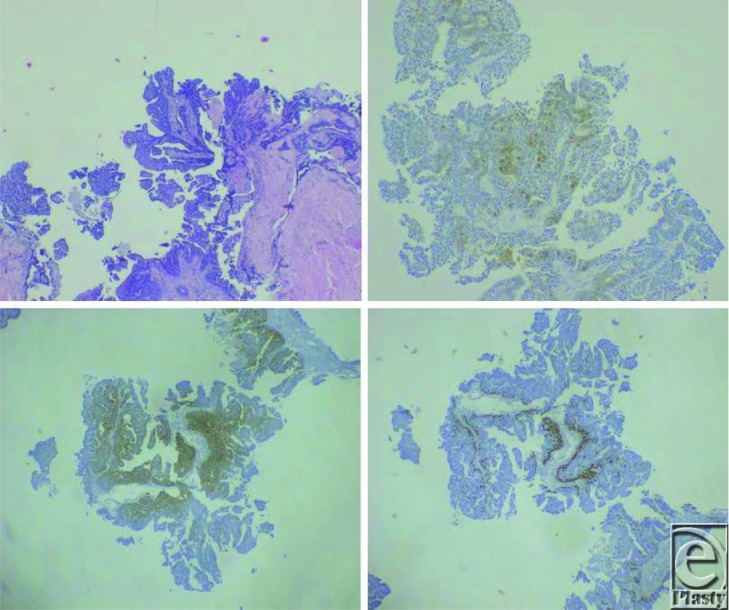
(*Top left*) Hematoxylin and eosin stains demonstrating clear papillary adenocarcinoma architecture. (*Top right*) Patchy positive s100 staining. (*Bottom left*) Cytokeratin 5 and 6 stains in conjunction with (*bottom right*) p63 stains confirming adnexal duct origin. Further TTF-1 and PAX-8 staining ruled out metastatic lesions from lung and thyroid.

Diagnosis can often be difficult, as they closely resemble their benign papillary adenomatous counterparts, showing fibrocollagenous stroma packed cells with prominent alveoli and focal squamous metaplasia with necrosis on histology.^6^ Given the rarity of primary adenocarcinomas of the digits, a thorough medical and oncological workup is mandatory to rule out a metastatic lesion from lung, thyroid, or breast malignancies. Figure 2 demonstrates the myriad of stains used to distinguish an ADPA from metastatic adenocarcinomas by looking at adnexal markers as well as those characteristic of lung and thyroid carcinomas.

Treatment of ADPA is wide surgical excision with possible partial or complete amputation of the digit involved, although what adequate margins constitute are not firmly described. Mohs' micrographic surgery has been described in some cases.^5^ There has been no official recommendation for adjuvant therapy due to the rarity of the cancer; however, it has been proposed that a sentinel node biopsy may provide beneficial information in identifying those at greater risk of metastasis. Adjuvant chemotherapy may be initiated in this population of patients despite overall poor responses.^4^^,^^5^

In the original study by Duke et al,^3^ it was found that only 5% of tumors recurred with surgical reexcision or amputation of the digit within 6 months, compared with 50% recurrence rate if not adequately reexcised.^2^ Metastasis rate has been estimated around 14% in patients with ADPA; therefore, there should be close follow-up with yearly chest radiographs looking for metastases, as 71% are found in the lung.^7^ Those found to have metastatic lesions died approximately 5 to 20 years after original diagnosis, indicating the range of possible outcomes.^8^

ADPAs are a very rare form of primary cancer found in the hand. Our patient had a characteristic clinical course of a slow-growing and painless mass that after an extensive oncological workup revealed was primary in origin. As previous studies have not been able to establish the efficacy of adjuvant therapy, surgical excision with possible amputation remains the principle treatment option. Despite favorable recurrence rates after excision, patients with ADPA need to be followed closely, as the metastasis rate is significant and can occur many years after surgery.
